# Subjective and Objective Consequences of Stress in Subjects with Subjectively Different Sleep Quality—A Cross-Sectional Study

**DOI:** 10.3390/ijerph18199990

**Published:** 2021-09-23

**Authors:** Beatrice Thielmann, Robin Sebastian Schierholz, Irina Böckelmann

**Affiliations:** Institute of Occupational Medicine, Medical Faculty, Otto von Guericke University, 39120 Magdeburg, Germany; r.schierholz@htp-tel.de (R.S.S.); irina.boeckelmann@med.ovgu.de (I.B.)

**Keywords:** heart rate variability, autonomic nervous system, sleep quality, stress, mental health

## Abstract

Background: Restful sleep plays an important role in long-term health and occupational safety. Heart rate variability (HRV) is used as stress indicator. The aim of this study was to determine whether HRV at rest or during sleep, as an objective indicator of stress, reflects subjectively assessed sleep quality. Methods: 84 subjects (37.3 ± 15.6 years) were classified into good sleepers and poor sleepers based on the results of the Pittsburgh Sleep Quality Index (PSQI). The cut-off value to distinguish between good and bad sleepers recommended by Buysse et al. 1989 is >5. Mental health status was determined using the 12-Item General Health Questionnaire (GHQ-12). A 24 h electrocardiogram (ECG) was recorded for HRV analysis (total and 6 h night phase). Results: The poor sleepers showed a significantly lower mental health status (*p* = 0.004). The multifactorial variance analysis of the total phase time parameters Min HR (*p* = 0.032, η^2^ = 0.056) and SI (*p* = 0.015, η^2^ = 0.072) showed significant interaction effects. In the 6h night phase, significant interaction effects were found for SDNN (*p* = 0.036, η^2^ = 0.065) and SD2 (*p* = 0.033, η^2^ = 0.067). In addition, there was a significant negative correlation between HRV and stress and a positive correlation between HRV and mental health. Conclusions: Although this study did not demonstrate a direct relationship between sleep quality and HRV, it was shown that there are important connections between sleep quality and mental health, and between HRV and mental health.

## 1. Introduction

Restful sleep is important for the performance of the employee, her or his mental well-being, and long-term health and work productivity [1]. The physiological importance of sleep for humans can be concluded from the fact that they spend about 1/3 of their lives asleep [2]. The U.S. National Sleep Foundation recommends seven to nine hours of sleep per day for adults 18 to 64 years old, and seven to eight hours of sleep per day for adults 65 years of age and older [3]. In addition to sleep duration, other aspects such as depth of sleep or sleep quality characterize healthy sleep [3].

The economic burden of poor sleep quality and sleep disorders is substantial [4,5]. Sleep problems lead to increased absenteeism from work due to illness [6]. However, sleep disorders are rarely documented as a cause of incapacity to work. Thus, they play a minor role in the incidence of inability to work [7]. In the long term, they increase the risk of occupational disability after an illness [8] and lead to early retirement [9]. They are also associated with an increased risk of occupational accidents [10]. This shows that healthy sleep is of particular importance for occupational medicine and thus for all workers. The occupational health practitioner should measure subjective and, if possible, objective quality of sleep.

Therefore, there is a high occupational healthcare relevance in the working world of those employees affected with sleep disorders. Not only occupational medicine uses methods to objectify stress. Heart rate (HR) and heart rate variability (HRV) measurement are well established in this regard. This measurement and analysis method is comfortable and non-invasive. It is established in out-patient and in-patient care, as well as in various research areas of medicine and natural sciences [11]. HR provides information about the degree of physical or psychological stress on the cardiovascular system in response to stress, whereas HRV provides additional information about the mechanisms of cardiovascular regulation [11]. HRV is considered to be the result of activation of different regulatory mechanisms of the organism to control cardiovascular homeostasis [12]. Additionally, under constant load HR undergoes a physiological variability that expresses inter alia the interaction of the two parts of the autonomic nervous system (ANS): the sympathetic and the parasympathetic nervous system. At rest and during low stress, parasympathetic predominates over sympathetic control, which is associated with higher variability of heart rhythm [11]. The different HRV parameters help to understand the interaction of sympathetic and parasympathetic nervous system in the regulation and control of the cardiovascular system during different types of demands [13]. The ANS is involved in stress regulation, as chronic stress has been associated with reduced HRV and reduced parasympathetic modulation [14]. HRV includes a large number of mathematically calculated parameters, which characterize the variance, rhythmicity or complexity of a time series of consecutive heart beats. These are defined as the normal-to-normal interval (NN) or R-peak-to-R-peak interval (RR) in the electrocardiogram (ECG). The established methods of HRV analysis are divided into methods of time domain, frequency domain and nonlinear analysis [11,15]. If parasympathetic activity should be evaluated, the parameter RMSSD (Root mean square of successive differences) is the parameter of choice and if total variability of the heart beat sequence is of interest, the parameter SDNN (Standard deviation of all normal-to-normal R–R intervals) [11]. Independent of the actual load, the HRV is influenced by a large number of modifiable and non-modifiable physiological and external factors, which play an important role in the assessment of HRV [11,16]. Thus, current guidelines should be used for interpretation [11,15]. Important parameters describing HR and HRV are shown in Table 1.

Various major epidemiological studies have shown that poor sleep quality and short sleep duration are associated with cardiovascular disease [17,18]. A pathophysiological mechanism for this could be changes in the ANS. Some studies have investigated this in healthy subjects. A study of 527 subjects showed that short sleep duration, poor sleep quality, and low sleep efficiency are associated with low values of the HF band of HRV (parasympathetic activity) [19]. Nocturnal awakening, sleep latency, lower sleep efficiency, and lower sleep duration are associated with higher LF/HF ratio (sympathetic/parasympathetic influences) [20]. Poor sleep quality is related to reduced HRV [21]. Short sleep duration correlates with higher mean HR and decreased HRV [19,20]. However, there are also studies that have not demonstrated HRV changes with regard to sleep restriction [22].

The focus of this work is on psychological stress. Work-related psychosocial stress is also associated with an increased risk of cardiovascular disease [23]. HRV can be used as an indicator of mental stress [11]. Work-related psychosocial stress is associated with increased values of Mean HR and LF/HF ratio in a study of 653 healthy male [24,25]. In addition, there are studies that could not demonstrate a sure relationship between work-related psychosocial stress and HRV [26,27]. Socioeconomic status should also be considered which is determined by social class, household income, ethnicity and education. It plays an important role in the development of mental disorders including anxiety and depression, and sleep parameters [28,29,30,31]. Socioeconomic status also influences allostatic load which is the chronic accumulation of stress over a lifetime [31].

Due to inconclusive previous study results, this study investigated the question to what extent HRV at rest or during sleep as an objective indicator of ANS reflects subjectively perceived sleep quality. Other factors such as sociodemographic and medical aspects as well as mental health were also included in the analysis. The following hypotheses were examined:

- With lower subjective sleep quality HRV decreases significantly, so that a reduction in vagal activity is recognizable.
- Poor sleep quality is associated with subjectively disturbed mental health measured with the 12-Item General Health Questionnaire (GHQ-12).
- There is a positive correlation between HRV and subjective mental health measured with the GHQ-12.

## 2. Materials and Methods

### 2.1. Subjects

Data from 84 subjects (mean age 37.3 ± 15.6 years, median 33 years, range 19–71 years) were included in the analysis, among them 42 female and 42 male subjects. On average, the female participants were 38.2 ± 14.5 years old (median 38.5 years, range 20–71 years) and the male participants 36.4 ± 16.7 years old (median 28 years, range 19–71 years). The sample was composed largely of workers who presented themselves at the occupational out-patient department for regular preventive medical examinations and voluntarily agreed to participate in the study. In addition, subjects were recruited via announcements at health days in companies or via private contacts. The data were collected in the time from September 2015 to February 2017.

### 2.2. Procedure

To participate in the study the subjects were invited to the Occupational Out-Patient Department of the Department of Occupational Medicine of the Medical Faculty of Otto von Guericke University Magdeburg. Written informed consent was obtained. The subjects completed a questionnaire and an activity protocol to note the activities in the following 24 h while wearing the ECG. After about 24 h the completed questionnaire, the protocol and the ECG were returned. After clinical evaluation of the ECG by a specialist doctor and HRV analysis an evaluation consultation was conducted with the subjects.

To exclude the influence of shift work on HRV [32], only subjects who had not been employed in shift work were included in the study. Further exclusion criteria relevant to HRV analysis were defined [11]: extrasystoles > 1% in total ECG recordings, ECG recording duration < 22 h, use of medication that affect heart rhythm, and physician-diagnosed diseases such as diabetes mellitus, untreated thyroid diseases and treated thyroid diseases with thyroid blood levels outside the normal range, cardiac diseases, nocturnal oxygen administration or supportive mechanical ventilation therapy, and diseases of the central or peripheral nervous system.

A detailed catalog of questions was prepared for the subjective data collection which included:

- Sociodemographic and health data,
- Pittsburgh Sleep Quality Index (PSQI) [33], and
- 12-Item General Health Questionnaire (GHQ-12) [34].

Objective sleep behavior has been assessed by measuring or calculating:

- Heart rate (HR) parameters, and
- Heart rate variability (HRV) parameters.

### 2.3. Sociodemographic and Health Data

Data regarding age, height and weight (for calculation of body mass index (BMI)), waist and hip circumference (for calculation of waist-to-hip ratio (WHR)) were collected, as well as information regarding the field’s interaction between work and private life, health, dietary habits and sports activities. Arterial systolic blood pressure (sBP) and diastolic blood pressure (dBP) were measured after a 3 to 5-min resting period with boso medicus PC, Jungingen, Germany. Sport activities were asked about, including how many years of continuous and regular weekly sport had been practiced.

### 2.4. Subjective Sleep Quality—Pittsburgh Sleep Quality Index (PSQI)

The Pittsburgh Sleep Quality Index (PSQI) is a self-report questionnaire used to identify subjective sleep quality over the past month [33]. The PSQI comprises 19 self-assessment questions and five questions for third-party assessment by a partner or roommate. The five questions of the external assessment are not included in the quantitative evaluation, but are used only as clinical information. The 19 items generate seven component scores: subjective sleep quality, sleep latency, sleep duration, habitual sleep efficiency, sleep disturbances, use of sleeping medication, and daytime dysfunction. Each of them can have a value from 0 to 3 points. The sum of the component scores yields the total score, which can range from 0 to 21 points. Higher scores indicate worse sleep quality. The cut-off value to differentiate between good and poor sleepers is >5 points [33].

In this study the subjects were divided into the two groups “good sleepers” and “poor sleepers”.

### 2.5. Health-Related Data—12-Item General Health Questionnaire (GHQ-12)

The 12-Item General Health Questionnaire (GHQ-12), a short version of the GHQ, measures general mental health status [34]. Unlike the original, the GHQ-12 in this study does not speak of “the last few weeks” [34], but of a time window of “the last four weeks”. It consists of 12 questions screening for recently experienced dysfunctional symptoms and behaviors. A four-point response scale is available (“better than usual”, “as usual”, “worse than usual”, and “much worse than usual”). In this study, the Likert-scoring (response scaling 0–1–2–3) and especially for the GHQ-12 recommended dichotomous GHQ-scoring (response scaling 0–0–1–1) was applied [35]. The sum of the dichotomous GHQ-scoring ranges from 0 to 12 points with a higher total score indicating greater mental health impairment. In this work a cut-off value for disturbed mental health of ≥5 points was used [36,37,38]. The GHQ-12 was used for factor analysis.

### 2.6. Heart Rate Variability Analysis—24-Hour Electrocardiogram (24h ECG)

ECG devices of the type medilog^®^ AR12 PLUS, SCHILLER, Baar, Switzerland were used. These are 3-channel ECG devices with automatic detection of the R peak, in which the sampling frequency was set to 1000 Hz. The raw data was processed, manually cleaned from artifacts and prepared for HRV analysis with the processing software medilog^®^ DARWIN2 Enterprise, SCHILLER, Baar, Switzerland. For the calculation of HRV parameters the program Kubios HRV version 2.0, University of Eastern Finland, Kuopio, Finland was used [39,40]. Only the parameters Stress Index (SI) and Coefficient of Variation (CV) were calculated in Microsoft Excel, Microsoft Corporation, Redmond, WA, USA using the formula of the SI (Equation (1)) [41]:

(1) $$\mathrm{SI}=\frac{\mathrm{AMo}}{2\times \mathrm{Mo}\times \mathrm{MxDMn}}$$

with Mo as mode value of the RR intervals, AMo as amplitude of the mode value and MxDMn as range of variation of the RR intervals, and using the formula of the CV (Equation (2)) [13]:

(2) $$\mathrm{CV}=\frac{\mathrm{SDNN}}{\mathrm{Mean}\;\mathrm{RR}}\times 100$$

On the one hand the total time of 24 h and on the other hand a six-hour night window was analyzed. Depending on when the subject went to rest, the night window starts at 11:00 p.m. if possible, but not after 01:00 a.m. For participants who rested for less than six hours during the recording period or who went to sleep after 01:00 a.m., no night window was produced. HRV parameters from time domain, frequency domain and nonlinear domain were calculated [11,41]. For this, the default settings [15,39] modified according to [42] were applied in Kubios HRV version 2.0 (Kubios, Kuopio, Finland). The HR and HRV parameters shown in Table 1 were examined in the context of this work. For some parameters, the total time (24 h) was examined only. The frequency domain was analyzed using Fast Fourier Transform (FFT).

### 2.7. Statistical Analysis

The statistical analysis of the data was performed with the statistical and analysis program IBM SPSS Statistics 24, IBM, Armonk, NY, USA. The following parameters were included in the sample size design: test = single factor ANOVA, number of groups = 2 (good and bad sleepers), type of power analysis = A priori, tails = Two, effect size η^2^ = 0.10 effect; alpha level α err prob = 0.05, and statistical power (1–*β* err prob) = 0.8. 37 subjects per group (74 in total) would be needed to obtain a significant result.

A significance level of 5% was defined. First, descriptive statistics were performed in which mean values and standard deviations (MW ± SD in tables) as well as medians and ranges were determined. To test for normal distribution, the Kolmogorov-Smirnov test was used. In case of normal distribution and interval-scaled data, the two-sample *t*-test for independent samples was applied. For ordinal-scaled or non-normally distributed interval-scaled variables, the Mann-Whitney U test was used. To counteract alpha error accumulation, Bonferroni correction was used for multiple mean comparisons. For categorical variables, Pearson’s χ^2^ test was used. For expected cell frequencies less than or equal to 5, Fisher’s exact test was applied. Multifactorial analyses of variance were conducted to test for interaction effects of multiple categorical variables. For significant main effects, additional Bonferroni-corrected post-hoc tests were performed provided that a factor had more than two expressions. Lastly, correlation analyses were performed to determine relationships between variables. Since at least one of the correlated variables was not normally distributed or only ordinally-scaled, Spearman’s rank correlation was used.

## 3. Results

### 3.1. Sociodemographic and Health Data as well as Classification of the Groups

The total sample was 84 subjects. Based on the result in the PSQI the subjects were divided into the two groups “good sleepers” (*n* = 53) and “poor sleepers” (*n* = 31) according to [33]. The good sleepers comprised 52.8% males and 47.2% females. There is a comparable distribution of both sexes in both groups (*p_χ^2^_* = 0.498). Good sleepers and poor sleepers were not different in sociodemographic and health data which is shown in Table 2. The subjects of both groups had normal BMI according to [43] and normal arterial blood pressure according to [44]. There was no significant difference in the distribution of occupation and general smoking status.

### 3.2. Subjective Sleep Quality and Mental Health

The average PSQI score was 5.5 ± 3.3. The PSQI score of the total sample is just above the cut-off value (Table 2). The poor sleepers showed higher and therefore worse scores in all components of PSQI. Since the total score is used for group classification *p*-values are not presented.

### 3.3. Results Regarding Heart Rate (HR) and Heart Rate Variability (HRV) Parameters (24 h and Six-Hour Night Window)

In the 24-h time frame the HR parameter Dynamics B showed a significant difference between both groups (*p* = 0.048), while all calculated HRV parameters were not significantly different (Table 3). Seven of the 84 subjects included in this evaluation (8.3%) went to sleep after 01:00 a.m., and 7 subjects (8.3%) had a rest period of less than six hours. These were not included in the analysis of the night window (from 11:00 p.m. to 05:00 a.m., if possible). Night phases shorter than 6 h or fragmented night phases are not recommended [42]. Consequently, just 70 subjects were included in the evaluation of the night window, of which 44 subjects (62.9%) were good sleepers and 26 subjects (37.1%) poor sleepers. No significant differences were found between the calculated HR and HRV parameters of the two groups in the night phase (Table 3).

### 3.4. Results of the Analysis of Variance

To examine HR and HRV parameters for interaction effects between mental health and subjective sleep quality, multifactorial analyses of variance were performed for the total 24-h period and for the six-hour night window. In addition to the group classification on the basis of the PSQI, a further factor was the classification according to the GHQ-12. The results are shown in Table 4.

In terms of the total time of 24 h there were significant interaction effects for the combined factors of PQSI and GHQ-12 only in the parameters Min HR (*F* = 4.767, *p* = 0.032, η^2^ = 0.056) and SI (*F* = 6.198, *p* = 0.015, η^2^ = 0.072). No significant main effect of the two individual factors with regard to the HR and HRV parameters was found.

At the six-hour night window the combined factors PSQI and GHQ-12 also yielded significant interaction effects for two parameters. These were the HRV parameters SDNN (*F* = 4.563, *p* = 0.036, η^2^ = 0.065) and SD2 (*F* = 4.756, *p* = 0.033, η^2^ = 0.067). There was no significant main effect of the two individual factors with regard to the HR and HRV parameters either.

### 3.5. Associations between Subjective Sleep Quality and General Mental Health as well as Heart Rate and Heart Rate Variability

**Figure 1:**
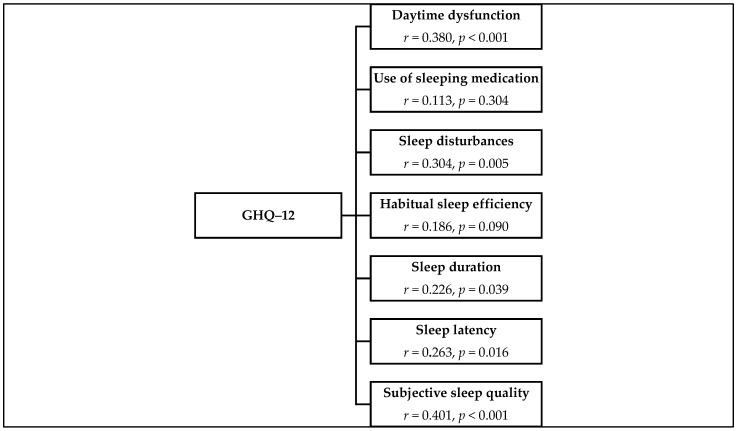
Correlations of GHQ-12 global score with PSQI component scores.

The global GHQ-12 score correlated significantly positively with the global PSQI score (*r* = 0.414, *p* < 0.001). Apart from the components use of sleeping medication and sleep efficiency the PSQI component scores had significant positive correlations with the GHQ-12 score. The results are shown in Figure 1.

The global PSQI score correlated negatively with the HR parameter Dynamics B (*r* = −0.239, *p* = 0.028), the HRV parameters SDNN (*r* = −0.269, *p* = 0.013), SD2 (*r* = −0.266, *p* = 0.014) and CV (*r* = −0.281, *p* = 0.009), and positively with the HR parameter Min HR (*r* = 0.265, *p* = 0.015) for the total time of 24 h. For the six-hour night window, no significant correlations of HR and HRV parameters with the global PSQI score were found. The results are demonstrated in Table 5.

For the total time of 24 h, three significant correlations of HR and HRV parameters with the GHQ-12 global score were found. The HRV parameters SDNN (*r* = −0.257, *p* = 0.018) and SD2 (*r* = −0.250, *p* = 0.022) were negatively correlated, and the HR parameter Min HR (*r* = 0.310, *p* = 0.004) was positively correlated with the GHQ-12 score. For the six-hour night window, there was one significant negative correlation of the HRV parameter SDNN with the global GHQ-12 score (*r* = −0.317, *p* = 0.008). Moreover, the HRV parameters RMSSD (*r* = −0.281, *p* = 0.019), pNN50 (*r* = −0.246, *p* = 0.040), SD1 (*r* = −0.280, *p* = 0.019), and SD2 (*r* = 0.303, *p* = 0.011) were correlated significantly negatively with the global GHQ-12 score.

## 4. Discussion

In this work, a possible relationship between the subjective rating of sleep quality and mental health and the objectively measured HR and HRV as indicators of stress was examined. For this purpose, the participants were divided into the two groups good sleepers and poor sleepers. The aim of the work was to assess the ability of HRV at rest or during sleep as an objective indicator of ANS to reflect subjectively perceived sleep quality. Only a few differences in HRV were found between the two groups. There was a significant positive correlation between HRV and mental health and a significant negative correlation between HRV and mental stress, respectively.

This study used a heterogeneous sample with equal numbers of male and female subjects. The age range was very large, with the youngest participant aged 19 and the oldest participant aged 71. Age on its own is a major factor influencing HRV [11,45]. The two groups based on the PSQI results did not differ significantly in age. The recorded sociodemographic and medical data (e.g., gender, BMI, blood pressure, exercising, smoking status) also showed no significant differences. This circumstance is an optimal condition for the evaluation of this study because comparable baseline prerequisites existed. Increased values of some of these factors are associated with poor sleep quality and short sleep duration [46,47]. Both groups did not differ significantly in the calculated HR and HRV parameters, neither for the total time of 24 h nor for the six-hour night window. In the multifactorial analyses of variance some interaction effects between subjective sleep quality and mental health in relation to some HR and HRV parameters were found. In the correlation analyses between subjective sleep quality and HR, as well as HRV, five significant correlations were found for the total time of 24 h. The subjectively perceived sleep quality of the 84 subjects in this study seemed to have little to no association with HRV, so that the balance of the sympathetic and parasympathetic nervous system did not appear to be significantly modified. Regarding the relationship between HRV and subjective sleep quality or sleep duration, there are relatively few studies on healthy subjects as in this study. A study of 199 healthy female subjects with similar mean age as the sample in this study found a significant negative relationship between sleep quality and the HRV parameter RMSSD for the examined day window [21]. There was indication of impaired balance of ANS in the form of decreased parasympathetic activity, but a different questionnaire was used to evaluate subjective sleep quality. The correlation existed in opposite directions for disturbed sleep and HRV during the working day. It was independently of demographic and behavioral confounders, but was only significant for the day window. Additionally, significant correlations for a day window were found for the HF band in a study of 29 healthy female subjects [48]. Interestingly in the context of intervention studies (poor sleep by forced sleep deprivation) results showed both a negative effect of sleep deprivation on HRV [49,50] and no significant effect on HRV [22]. The fact that in this study no significant associations between HRV of the night window and subjective sleep quality were found is consistent with results of other authors [48,51]. Possible explanations are fluctuations of HR and HRV over the different sleep stages [48,51] and over different lengths of periods of the same sleep phase during the night. This leads physiologically to a high heterogeneity of the HRV of one night and relatively low reliability over several nights [48]. During REM sleep, HR sometimes increases even higher than during awareness, and HRV is reduced [52]. Subjective sleep quality on its own seems to have a difficultly measurable effect on HRV during sleep as long as there is no manifest sleep disorder. A manifest sleep disorder such as insomnia leads to significantly increased values of the LF band and decreased values of the HF band during sleep compared to healthy sleepers [53]. Other sleep disorders also cause changes in HRV [51]. In the correlation analyses performed for the total 24-h period and the six-hour night window, significant negative correlations (except for the HR parameter Min HR) were found between HR and HRV parameters and the GHQ-12 score. It is notable that several significant correlations (SDNN, RMSSD, pNN50, SD1, SD2) were found for the six-hour night window, whereas only three significant correlations (Min HR, SDNN, SD2) were found for the 24-h total time. This could be caused by the fact that different activities took place in the 24-h period. A reduction in HRV is caused by mental illnesses such as anxiety disorders, post-traumatic stress disorder or depression [11]. It cannot be ruled out that subjects suffered from mental illness during the study period. However, it can be assumed that the more impaired mental health was, that is to say the more the subject’s mental health was close to a pathological condition such as depression or anxiety disorder, the lower the HRV was.

An important aspect for good mental and physical health is sufficient and restful sleep [54]. Further results of this study reflect this relationship. Mental health assessed with the GHQ-12 [34] differed very significantly between good sleepers and poor sleepers. These results were also confirmed in the correlation analyses in which mental health correlated significantly positively with subjective sleep quality measured by the PSQI. Given the cross-sectional design of this study no inference of causality is possible. It is both possible that subjective sleep quality had an impact on mental health as well as that mental health had an influence on subjective sleep quality. A bidirectional relationship is most likely since on the one hand disturbed sleep is symptom of various mental illnesses and conditions [55], but on the other hand it also causes burnout [56] and mental illness [57]. Short sleep duration is associated with burnout [58], depression [59], and suicidality [60]. Social and work-related stress can affect sleep quality [61]. Constant accessibility in the modern world of work has advantages and disadvantages for the health status of the employee. Extended availability can lead to additional workloads outside regular working hours and to fragmentation of individual areas of life, which has consequences for stress and recovery [62]. Systematic reviews and meta-analyses found that socioeconomic status also influenced sleep parameters, with lower socioeconomic status being associated with disturbed mental health and poorer sleep parameters [28,31,63,64]. Lower socioeconomic status was observed to be associated with lower total sleep duration, longer sleep latency, greater sleep fragmentation and higher sleep onset variability [64].

Limitations of this study should be considered. Since a cross-sectional study design was used, no conclusions about the causality of the found associations can be made. For this it would be necessary and interesting to conduct a longer longitudinal study with regular follow-up investigations. Furthermore, the study population did not constitute a representative sample with a limited number of participants. A part of these were subjects who participated in health days, which could indicate that they lived a particularly health conscious lifestyle leading to a possible selection bias in the recruitment of participants. These people may show increased health care for themselves. The PSQI was used to assess subjective sleep over the last four weeks. Longer-term sleep problems were not enquired apart from nocturnal oxygen application and supportive mechanical ventilation therapy as exclusion criteria. Diagnosed sleep disorders and mental disorders were not queried in this study either. It was also not specified which sleeping medication was used if the subject reported in the PSQI that he or she took sleeping medication. It is possible that the subject reported no sleep problems in the last four weeks in the PSQI, but that he or she had sleep problems in the night the HRV analysis was conducted. HRV during sleep can be influenced by physiological stress experienced immediately before going to sleep [65]. Indications of any consciously experienced sleep problems or possible stress factors were obtained from the activity protocol. It is also possible that sleep and general condition of the subject over the recording period were influenced by the measuring device (ECG device, electrodes, wires). Certain wireless chest strap systems could be considered equivalent and used if necessary [66]. Despite prior instructions it cannot be ruled out with certainty that the subjects did not fail to perform certain activities, e.g., doing sports, and then perhaps concealed them in the activity log. It also seems to make sense to conduct interdisciplinary studies, such as with pneumology. In this way, polysomnographic examinations could also be used as objective measures of sleep since polysomnography is not a typical occupational health method. The GHQ-12 also examines only the mental health status of the last four weeks. Apart from the question about psychiatric medication in the questionnaire no further psychopathological examination was performed. A possibility of distortion of the described associations regarding mental health and sleep quality could be that a subject with impaired mental health or poor subjective sleep quality might have a negative and pessimistic general attitude and therefore tends to evaluate the respective other factor consciously or unconsciously worse than it actually is and a subject with good mental health or good subjective sleep quality possibly could have a positive and optimistic general attitude and evaluates the respective other factor intentionally or unintentionally better than it actually is. Of course, it should not be forgotten that stress itself can have a significant impact on sleep and HRV. Data on socioeconomic status such as social class, household income, ethnicity and education were not determined. As these characteristics can play an important role in the development of mental disorders, they should be taken into account in future studies.

On the other hand, one of the strengths of this work is the method of recording the HRV data. Many studies with similar examined problems are limited to short recording windows and often use a relatively low sampling frequency. In this work three-channel ECG devices with automatic detection of the R peaks, a set sampling frequency of 1000 Hz and a recording duration of 24 h were used. This increased accuracy in detecting the intervals between individual cardiac actions and minimizes problems with the detection of artifacts.

## 5. Conclusions

In summary, although no direct relationship between sleep quality and HRV was found, it was confirmed that there are important associations between sleep quality and mental health as well as between HRV and mental health. The results emphasize the importance of restful sleep for mental well-being and vice versa. In this way, they indirectly show possibilities of preventive interventions for psychosocial stress and sleep problems.

## Figures and Tables

**Table 1:** Overview and definitions of examined heart rate (HR) and heart rate variability (HRV) parameters.

**Heart rate (HR)**		
Mean HR [1/min]		Mean heart rate
Max HR [1/min] ^†^		Maximum heart rate
Min HR [1/min] ^†^		Minimum heart rate
Dynamics A [1/min] ^†^		Difference between Mean HR of day and Mean HR of sleep
Dynamics B [1/min] ^†^		Difference between Max HR and Min HR
**Heart rate variability (HRV)**		
Time domain parameters	Mean RR [ms]	Average R–R interval duration in a measurement
	SDNN [ms]	Standard deviation of all normal-to-normal R–R intervals
	RMSSD [ms]	Root mean square of successive differences
	pNN50 [%]	Percentage of successive NN intervals that differ by more than 50ms
	CV [%] ^†^	Coefficient of Variation
	HRV Tri Index	Integral of the density of the RR interval histogram divided by its height
	TINN [ms]	Baseline width of the RR interval histogram
	SI ^†^	Stress Index
Frequency domain parameters	LF nu	Relative power of the low-frequency band (0.04–0.15 Hz) in normal units
	HF nu	Relative power of the high-frequency band (0.15–0.4 Hz) in normal units
	LF/HF	Ratio of LF-to-HF power
Nonlinear domain parameters	SD1 [ms]	Poincaré plot standard deviation perpendicular the line of identity
	SD2 [ms]	Poincaré plot standard deviation along the line of identity
	Alpha 1	Detrended fluctuation analysis which describes short-term fluctuations
	Alpha 2	Detrended fluctuation analysis which describes long-term fluctuations

Notes: ^†^ indicates parameter for which only the total time (24 h) was examined.

**Table 2:** Sociodemographic and health data as well as sleep quality and mental health of both groups.

Variables	Good Sleepers	Poor Sleepers	Total	Significance *p* ^a b^
Sociodemographic and health data AV ± SD median (range)				
Age [years]	35.2 ± 14.3 29 (19–64)	40.9 ± 17.2 47 (19–71)	37.3 ± 15.6 33 (19–71)	0.195
BMI [kg/m^2^]	24.37 ± 3.94 23.46 (17.63–38.57)	24.84 ± 4.20 24.13 (19.33–34.36)	24.55 ± 4.02 23.89 (17.63–38.57)	0.680
WHR	0.88 ± 0.11 0.89 (0.68–1.29)	0.89 ± 0.11 0.88 (0.70–1.20)	0.89 ± 0.11 0.89 (0.68–1.29)	0.637
sBP [mmHg]	125.9 ± 10.8 125 (90–158)	124.4 ± 15.6 121 (99–167)	125.3 ± 12.7 124 (90–167)	0.164
dBP [mmHg]	79.3 ± 8.7 80.5 (60–105)	78.8 ± 11.0 78 (57–102)	79.1 ± 9.6 79 (57–105)	0.415
Sport [times/week]	2.3 ± 1.9 2 (0–6)	1.8 ± 2.0 1 (0–8)	2.1 ± 2.0 2 (0–8)	0.251
Sport [years]	12.0 ± 12.2 10 (0–45)	8.7 ± 8.9 6 (0–30)	10.8 ± 11.1 10 (0–45)	0.357
Sociodemographic and health data n (%)				
Female	25 (59.5)	17 (40.5)	42 (50)	0.498 ^c^
Male	28 (66.7)	14 (33.3)	42 (50)	

Occupation—intellectual	40 (75.5)	23 (74.2)	63 (75.0)	0.424 ^d^
Occupation—physical	5 (9.4)	1 (3.2)	6 (7.1)	
Occupation—intellectual/physical	8 (15.1)	6 (19.4)	14 (16.7)	
Occupation—pensioner	0 (0.0)	1 (3.2)	1 (1.2)	

Smoker—former	8 (15.4)	7 (23.3)	15 (18.3)	0.596 ^d^
Smoker—current	4 (7.7)	1 (3.3)	5 (6.1)	
Non-smoker	40 (76.9)	22 (73.3)	62 (75.6)	
Sleep quality (PSQI) and mental health (GHQ-12) AV ± SD median (range)				
PSQI—Total score	3.5 ± 1.3 4 (0–5)	9.0 ± 2.6 8 (6–14)	5.5 ± 3.3 5 (0–14)	<0.001
GHQ-12—Total score	9.1 ± 3.7 9 (3–18)	12.9 ± 6.1 11 (7–26)	10.5 ± 5.0 9 (3–26)	0.004
Mental health (GHQ-12) n (%)				
GHQ-12—stable	51 (96.2)	22 (71.0)	73 (86.9)	0.002 ^d^
GHQ-12—impaired	2 (3.8)	9 (29.0)	11 (13.1)	

Notes: AV = Average, SD = Standard deviation. WHR = Waist-to-hip ratio. sBP/dBP = Arterial systolic/diastolicblood pressure. a = *t*-test, b = Mann-Whitney U test, c = Pearson’s χ^2^ test, d = Fisher’s exact test.

**Table 3:** Comparison of HR and HRV parameters in the 24-h time frame and six-hour night phase of both groups.

	24 h				6-h Night Phase			
	Good Sleepers	Poor Sleepers	Total	Significance	Good Sleepers	Poor Sleepers	Total	Significance
HR and HRV Parameters	AV ± SD Median (Range)			*p* ^ab^	AV ± SD Median (Range)			*p* ^a b^
Mean HR [1/min]	77.56 ± 8.18 76.9 (60.5–101.6)	77.36 ± 8.25 75.5 (65.1–96.0)	77.49 ± 8.15 75.6 (60.5 ± 101.6)	0.753	62.74 ± 6.49 61.8 (49.5–82.7)	68.53 ± 26.14 62.5 (51.3 ± 76.3)	62.87 ± 6.63 62.2 (49.5–82.7)	0.833
Max HR [1/min] ^†^	150.62 ± 22.03 153 (104–218)	143.06 ± 20.17 139 (111–186)	147.83 ± 21.55 146.5 (104–218)	0.121				
Min HR [1/min] ^†^	44.60 ± 6.50 44 (33–67)	46.81 ± 6.02 47 (36–62)	45.42 ± 6.38 45 (33–67)	0.068				
Dynamics A [1/min] ^†^	19.62 ± 6.78 19 (9–43)	18.55 ± 4.96 19 (9–28)	19.23 ± 6.16 19 (9–43)	0.444				
Dynamics B [1/min] ^†^	106.02 ± 23.09 107 (58–176)	96.26 ± 18.45 96 (61–124)	102.42 ± 21.90 100 (58–176)	0.048				
Mean RR [ms]	816.90 ± 88.72 811.9 (600.9–1045.8)	812.59 ± 84.53 823.9 (651.2–955.0)	815.31 ± 86.71 817.0 (600.9 ± 1045.8)	0.827	980.31 ± 101.24 982.6 (729.1–1251.2)	973.43 ± 108.31 969.4 (791.6–1182.7)	977.75 ± 103.20 976.8 (729.1–1251.2)	0.790
SDNN [ms]	163.08 ± 41.20 165.2 (77.1–258.7)	148.85 ± 30.57 145.3 (86.7 ± 213.5)	157.82 ± 38.06 158.8 (77.1–258.7)	0.098	110.17 ± 37.72 115.6 (50.1–214.0)	98.53 ± 26.14 100.6 (57.3–168.9)	105.84 ± 35.16 109.4 (50.1–214.0)	0.123
RMSSD [ms]	46.25 ± 27.01 36.5 (10.2–153.2)	35.76 ± 15.19 32.0 (17.0–74.4)	42.38 ± 23.80 34.6 (10.2–153.2)	0.098	59.01 ± 34.15 55.2 (15.8–165.5)	45.55 ± 21.08 43.0 (16.5–97.0)	54.01 ± 30.51 47.3 (15.8–165.5)	0.148
pNN50 [%]	17.51 ± 14.93 14.4 (0.3–68.4)	12.08 ± 10.06 9.6 (1.3–37.2)	15.51 ± 23.80 11.1 (0.3–68.4)	0.133	27.41 ± 20.66 30.9 (0.9–70.1)	20.13 ± 15.51 18.4 (1.2–55.3)	24.71 ± 19.13 20.6 (0.9–70.1)	0.160
HRV Tri Index	45.51 ± 14.20 41.75 (17.09–75.96)	44.41 ± 11.14 44.80 (22.92–67.75)	45.11 ± 13.09 42.36 (17.09–75.96)	0.967	27.33 ± 11.19 25.78 (11.70–57.91)	23.56 ± 6.68 23.01 (13.87–37.28)	25.93–9.88 24.85 (11.70–57.91)	0.082
TINN [ms]	574.53 ± 70.16 575 (395–830)	575.48 ± 67.93 570 (440–735)	574.88 ± 68.93 570 (395–830)	0.952	345.00 ± 59.46 332.5 (255–520)	345.38 ± 66.79 342.5 (170–450)	345.14 ± 61.80 340 (170–520)	0.980
CV [%] ^†^	19.87 ± 4.29 20.20 (11.24–35.09)	18.29 ± 3.01 18.81 (12.70 ± 24.58)	28.54 ± 13.88 19.47 (11.24–35.09)	0.074				
SI ^†^	26.94 ± 14.18 25.86 (2.67–82.65)	31.26 ± 13.13 27.93 (14.04–59.90)	28.54 ± 13.88 26.78 (2.67–82.65)	0.116				
LF nu	69.10 ± 13.01 72.5 (31.6−89.2)	70.41 ± 11.02 73.0 (36.0−87.2)	69.58 ± 12.26 72.8 (31.6−89.2)	0.799	63.31 ± 15.03 65.5 (31.2–91.3)	66.04 ± 13.34 69.2 (40.4–87.8)	64.32 ± 14.39 67.3 (31.2–91.3)	0.446
HF nu	30.90 ± 13.01 27.5 (10.8−68.4)	29.59 ± 11.02 27.0 (12.8−64.0)	30.42 ± 12.26 27.2 (10.8−68.4)	0.799	36.70 ± 15.03 34.5 (8.7–68.8)	33.96 ± 13.34 30.8 (12.2–59.6)	35.68 ± 14.39 32.7 (8.7–68.8)	0.446
LF/HF	2.89 ± 1.81 2.63 (0.46−8.25)	2.81 ± 1.35 2.70 (0.56−6.80)	2.86 ± 1.65 2.67 (0.46−8.25)	0.795	2.34 ± 1.88 1.91 (0.45–10.44)	2.50 ± 1.67 2.25 (0.67–7.23)	2.40 ± 1.79 2.06 (0.45–10.44)	0.504
SD1 [ms]	32.70 ± 19.09 25.8 (7.2–108.3)	25.29 ± 10.75 22.6 (12.0–52.6)	29.97 ± 16.83 24.5 (7.2–108.3)	0.099	41.74 ± 24.16 39.0 (11.2–117.1)	32.22 ± 14.91 30.4 (11.6–68.6)	38.20 ± 21.58 33.5 (11.2–117.1)	0.150
SD2 [ms]	227.89 ± 56.70 233.2 (108.8–364.4)	208.82 ± 42.62 204.5 (121.9–300.8)	220.85–52.50 223.1 (108.8–364.4)	0.109	149.48 ± 49.55 155.8 (67.2–285.2)	135.24 ± 35.13 138.8 (79.3–228.8)	144.19 ± 45.00 149.8 (67.2–285.2)	0.203
Alpha 1	1.29 ± 0.17 1.31 (0.75–1.61)	1.30 ± 0.16 1.34 (0.75–1.53)	1.29 ± 0.16 1.31 (0.75–1.61)	0.758	1.14 ± 0.22 1.16 (0.71–1.54)	1.18–0.19 1.22 (0.73–1.52)	1.16 ± 0.21 1.18 (0.71–1.54)	0.453
Alpha 2	1.02 ± 0.08 1.03 (0.76–1.22)	1.03 ± 0.08 1.05 (0.85–1.16)	1.02 ± 0.08 1.04 (0.76–1.22)	0.727	0.98 ± 0.07 0.99 (0.79–1.13)	1.00 ± 0.10 1.01 (0.83–1.20)	0.99 ± 0.09 1.00 (0.79–1.20)	0.559

Notes. ^†^: Parameter for which only the total time (24 h) was examined. a = *t*-test, b = Mann-Whitney U test.

**Table 4:** Interaction effects between classification by sleep group (PSQI) and GHQ-12 for HR and HRV parameters of total time (24 h) and six-hour night window.

		24 h			6-h Night Phase		
HR and HRV Parameters		*F*	*p*	η^2^	*F*	*p*	η^2^
Mean HR [1/min]	PSQI	1.547	0.217	0.019	2.017	0.160	0.030
	GHQ-12	0.299	0.586	0.004	2.311	0.133	0.034
	PSQIxGHQ-12	2.428	0.123	0.029	3.091	0.083	0.045
Max HR [1/min] ^†^	PSQI	0.697	0.406	0.009			
	GHQ-12	0.129	0.721	0.002			
	PSQIxGHQ-12	0.012	0.913	0.000			
Min HR [1/min] ^†^	PSQI	0.936	0.336	0.012			
	GHQ-12	2.053	0.156	0.025			
	PSQIxGHQ-12	4.767	0.032	0.056			
Dynamics A [1/min] ^†^	PSQI	0.247	0.620	0.003			
	GHQ-12	0.071	0.790	0.001			
	PSQIxGHQ-12	0.047	0.829	0.001			
Dynamics B [1/min] ^†^	PSQI	0.307	0.581	0.004			
	GHQ-12	0.590	0.445	0.007			
	PSQIxGHQ-12	0.267	0.607	0.003			
Mean RR [ms]	PSQI	1.220	0.273	0.015	1.342	0.251	0.020
	GHQ-12	0.419	0.519	0.005	1.509	0.224	0.022
	PSQIxGHQ-12	2.497	0.118	0.030	2.305	0.134	0.034
SDNN [ms]	PSQI	0.048	0.827	0.001	0.937	0.337	0.014
	GHQ-12	0.849	0.360	0.010	2.174	0.145	0.032
	PSQIxGHQ-12	1.816	0.182	0.022	4.563	0.036	0.065
RMSSD [ms]	PSQI	0.000	0.994	0.000	0.020	0.888	0.000
	GHQ-12	0.549	0.461	0.007	1.231	0.271	0.018
	PSQIxGHQ-12	1.773	0.187	0.022	1.932	0.169	0.028
pNN50 [%]	PSQI	0.095	0.758	0.001	0.282	0.597	0.004
	GHQ-12	0.814	0.370	0.010	1.389	0.243	0.021
	PSQIxGHQ-12	2.550	0.114	0.031	2.979	0.089	0.043
HRV Tri Index	PSQI	0.459	0.500	0.006	0.458	0.501	0.007
	GHQ-12	0.464	0.498	0.006	1.730	0.193	0.026
	PSQIxGHQ-12	1.112	0.295	0.014	3.618	0.062	0.052
TINN [ms]	PSQI	0.106	0.745	0.001	0.202	0.654	0.003
	GHQ-12	1.373	0.245	0.017	0.434	0.512	0.007
	PSQIxGHQ-12	0.000	0.996	0.000	0.176	0.676	0.003
CV [%] ^†^	PSQI	0.024	0.877	0.000			
	GHQ-12	0.698	0.406	0.009			
	PSQIxGHQ-12	0.860	0.357	0.011			
SI ^†^	PSQI	1.157	0.285	0.014			
	GHQ-12	0.547	0.462	0.007			
	PSQIxGHQ-12	6.198	0.015	0.072			
LF nu	PSQI	0.292	0.590	0.004	0.093	0.761	0.001
	GHQ-12	0.332	0.566	0.004	0.003	0.955	0.000
	PSQIxGHQ-12	0.042	0.838	0.001	0.047	0.829	0.001
HF nu	PSQI	0.292	0.590	0.004	0.093	0.761	0.001
	GHQ-12	0.332	0.566	0.004	0.003	0.955	0.000
	PSQIxGHQ-12	0.042	0.838	0.001	0.047	0.829	0.001
LF/HF	PSQI	0.093	0.761	0.001	0.268	0.606	0.004
	GHQ-12	0.416	0.521	0.005	0.042	0.839	0.001
	PSQIxGHQ-12	0.150	0.699	0.002	0.137	0.712	0.002
SD1 [ms]	PSQI	0.000	0.996	0.000	0.020	0.888	0.000
	GHQ-12	0.554	0.459	0.007	1.226	0.272	0.018
	PSQIxGHQ-12	1.776	0.186	0.022	1.938	0.169	0.029
SD2 [ms]	PSQI	0.056	0.813	0.001	1.124	0.293	0.017
	GHQ-12	0.842	0.361	0.010	2.200	0.143	0.032
	PSQIxGHQ-12	1.783	0.186	0.022	4.756	0.033	0.067
Alpha 1	PSQI	0.037	0.849	0.000	0.012	0.912	0.000
	GHQ-12	0.001	0.974	0.000	0.390	0.534	0.006
	PSQIxGHQ-12	0.300	0.585	0.004	0.352	0.555	0.005
Alpha 2	PSQI	0.852	0.359	0.011	0.058	0.810	0.001
	GHQ-12	0.723	0.398	0.009	1.085	0.301	0.016
	PSQIxGHQ-12	1.797	0.184	0.022	0.305	0.583	0.005

Notes. ^†^: Parameter for which only the total time (24 h) was examined.

**Table 5:** Correlations of PSQI and GHQ-12 global scores with HR and HRV parameters of total time (24 h) and six-hour night window.

		PSQI		GHQ-12	
HR and HRV Parameters		ECG—24 h	ECG—6-h Night Phase	ECG—24 h	ECG—6-h Night Phase
Mean HR [1/min]	*r* *p*	0.018 0.874	−0.107 0.334	0.112 0.309	0.223 0.063
Max HR [1/min] ^†^	*r* *p*	−0.202 0.065		−0.021 0.847	
Min HR [1/min] ^†^	*r* *p*	0.265 0.015		0.310 0.004	
Dynamics A [1/min] ^†^	*r* *p*	−0.097 0.382		−0.110 0.320	
Dynamics B [1/min] ^†^	*r* *p*	−0.239 0.028		−0.106 0.336	
Mean RR [ms]	*r* *p*	−0.048 0.666	0.133 0.227	−0.133 0.226	−0.231 0.055
SDNN [ms]	*r* *p*	−0.269 0.013	0.055 0.616	−0.257 0.018	−0.317 0.008
RMSSD [ms]	*r* *p*	−0.155 0.158	0.076 0.493	−0.179 0.104	−0.281 0.019
pNN50 [%]	*r* *p*	−0.154 0.161	0.080 0.472	−0.160 0.145	−0.246 0.040
HRV Tri Index	*r* *p*	−0.083 0.451	0.037 0.737	−0.103 0.352	−0.216 0.073
TINN [ms]	*r* *p*	−0.088 0.426	−0.055 0.619	−0.118 0.286	−0.055 0.652
CV [%] ^†^	*r* *p*	−0.281 0.009		−0.210 0.056	
SI ^†^	*r* *p*	0.184 0.094		0.124 0.263	
LF nu	*r* *p*	−0.013 0.909	−0.069 0.535	−0.005 −0.965	0.151 0.212
HF nu	*r* *p*	0.013 0.909	0.069 0.535	0.005 0.965	−0.151 0.212
LF/HF	*r* *p*	−0.012 0.916	−0.069 0.534	−0.005 0.967	0.150 0.216
SD1 [ms]	*r* *p*	−0.155 0.160	0.075 0.496	−0.179 0.103	−0.280 0.019
SD2 [ms]	*r* *p*	−0.266 0.014	0.057 0.604	−0.250 0.022	−0.303 0.011
Alpha 1	*r* *p*	−0.004 0.973	−0.137 0.212	−0.007 0.947	0.176 0.146
Alpha 2	*r* *p*	−0.006 0.954	0.134 0.224	−0.013 0.909	0.115 0.345

Notes. ^†^: Parameter for which only the total time (24 h) was examined.

## Data Availability

The data can be requested from the authors.
